# Unveiling the antifungal potential of extracts in leaves and branches from *Nicotiana glauca* for wood biofungicides

**DOI:** 10.1038/s41598-026-42531-x

**Published:** 2026-03-27

**Authors:** Mohamed Z. M. Salem, Abeer A. Mohamed, Mohammed A. A. Elshaer, Mohamed A. M. Abd-Elraheem, Zakaria H. Saad, Maisa M. A. Mansour, Mervat EL-Hefny

**Affiliations:** 1 Forestry and Wood Technology Department, Faculty of Agriculture (El- Shatby), Alexandria University, Alexandria, 21545 Egypt; 2 Plant Pathology Research Institute, Agriculture Research Center (ARC), Alexandria, 21616 Egypt; 3 Agriculture Biochemistry Department, Faculty of Agriculture for Boys, Al- Azhar University, Sadat City, Egypt; 4 Agriculture Biochemistry Department, Faculty of Agriculture, Al-Azhar University, Cairo, Egypt; 5 Organic Materials Conservation Department, Faculty of Archaeology, Cairo University, Giza, 12613 Egypt; 6 Department of Floriculture, Ornamental Horticulture and Garden Design, Faculty of Agriculture (El-Shatby), Alexandria University, Alexandria, 21545 Egypt

**Keywords:** Nicotiana glauca extracts, HPLC, GC-MS, Anabasine, Antifungal activity, Tobacco tree, Wood preservation, Biochemistry, Biological techniques, Biotechnology, Drug discovery, Microbiology, Plant sciences

## Abstract

Wild-growing medicinal plants are a rich source of bioactive compounds, which serve as antimicrobial agents for various medicinal, pharmaceutical, and wood preservation uses. In this study, branches and leaves from *Nicotiana glauca* Graham, a wild plant from Egypt, were used to extract bioactive compounds using the ethanol solvent. The ethanol extracts (EEs) were analyzed for their chemical components using HPLC and GC-MS. The EEs were applied to *Fagus sylvatica* L. wood to evaluate their activity against the growth of three fungi, namely *Phoma glomerata*, *Fusarium circinatum*, and *Pythium tardicrescens*. By HPLC analysis, the abundant phenolic and flavonoid compounds in EE from the branches were rutin (1529.37 µg/g dry extract), quercetin (856.96 µg/g dry extract), and gallic acid (813.79 µg/g dry extract). The *N. glauca* leaf EE contained high amounts of rutin (23364.18 µg/g dry extract), chlorogenic acid (3136.67 µg/g dry extract), gallic acid (1133.30 µg/g dry extract), and coumaric acid (1066.13 µg/g dry extract). The GC-MS analysis of the branches EE showed the abundant compounds methyl oleate (19.39%), oleic acid (17.09%), 9-octadecenal (15.65%), methyl palmitate (14.08%), and methyl 12,13-tetradecadienoate (8.99%). In the leaves EE, the primary compounds were anabasine (11.44%), palmitic acid (11.29%), oleic acid (10.96%), hydnocarpic acid (8.34%), and hexahydrofarnesyl acetone (6.76%). The EEs at 1000 µg/mL exhibited the best activity against the growth of *P. glomerata*, *P. tardicrescens*, and *F. circinatum* with fungal inhibition percentage (FIP) values of (35.92% and 27.77%), (58.15% and 47.41%), and (55.55 and 55.18%), respectively. The MICs with the branch and leaf extract ranged from 15.6 to 250 µg/mL with all fungal isolates. This study offers significant and important applications of *Nicotiana glauca* as a source of natural extracts for wood-biofungicides application against some fungi isolated from diseased branches and roots of *Pinus halepensis*.

**Supplementary Information:**

The online version contains supplementary material available at 10.1038/s41598-026-42531-x.

## Introduction

The tree tobacco, *Nicotiana glauca* Graham, is a noxious invasive fast-growing evergreen perennial shrub, belonging to the family Solanaceae^1,2^. It is well known for its harmful impact on biodiversity, competing with native plants using various strategies. Allelochemical activity, or the employment of chemicals to dominate and compete with other species, is one of their strategies. This plant, however, occurs mostly in warm areas due to its sensitivity to frost^3^. The invasive plant *N. glauca* grows in open, disturbed areas such as roadsides, creek lines, dry riverbeds, and wastelands^4^. The whole plant has been used as anodyne, a remedy for boils, piles, sores, and wounds^5,6^.

The preliminary phytochemical analysis of extracts of *N. glauca* showed that varying amounts of alkaloids, steroids, tannins, flavonoids, and saponins were present in the extracts of the leaves, stems, flowers, and roots^1,7^. The preliminary phytochemical screening has indicated that leaves and flowers of *N. glauca* contain a higher concentration of flavonoids and other phytochemicals compared to the stems and roots. The total alkaloidal content and its specific composition compound, anabasine, can also vary across different parts of the plant^8^. The methanol extracts from roots, stem, leaves, flowers, seeds, and bark of *N. glauca* contain high levels of alkaloidal fraction with anti-inflammatory and antioxidant activities^9^. Phenols and tannins are known to act as allelochemical compounds in *N. glauca*^10^, which is presented in high concentrations in the aqueous extracts from leaves and flowers^11^. The phenolic compounds in *N. glauca* are expected to act as allelochemicals^10,12^. Another study observed a highly significant variation in the phenolic content among the studied plants of *N. glauca* from different habitats^13^.

*N. glauca* is highly toxic to humans and animals, and all parts of the plant are extremely poisonous due to the presence of anabasine alkaloid^2,14,15^. The anti-neovascularization effect of scopoletin, an active principal extract from *N. glauca*, and its antitumorigenic activity on human tumors in xenograft models was reported^16^. *N. glauca* is well known as a toxic plant^11,14^; however, it has been used traditionally in medicine, where warmed leaves are applied to the head to relieve headache^17^, on the throat to relieve a sore throat, and put on shoes for painful feet. Moreover, the plant has been used as an insecticide^18^. *N. glauca* extracts were observed to have several biological activities, such as antimicrobial, anti-inflammatory, hypoglycemic potential, cytotoxic, and antioxidant^8,19–21^. The extracts from shoots and leaves of *N. glauca* showed antimicrobial activity against several bacterial and fungal isolates^1,22^.

To the best of our knowledge, there are no reports about the application of extracts from *N. glauca* to combat the fungal growth over organic materials such as wood. Therefore, to add value to this plant, the extracts can be used as a natural product for preserving wood and other organic materials. As our previous work used natural extracts as wood protection agents against fungal growth. For example, when 1% recoverable extract from the hydrodestillation of *Callistemon viminalis* (Sol. ex Gaertn.) G.Don essential oil was applied to linen and oakwood textile samples; the maximum inhibition value against the growth of *A. fumigatus* was 99.26%^23^. The painted wood from *Ficus sycomorus* L. treated with 100 µL/mL of the essential oil from *Thuja orientalis* L. aerial parts showed good antifungal activity against the growth of *Fusarium culmorum* and *Aspergillus niger*^24^. The maximum level of inhibition to *F. solani* was observed when the methanol extract from *Syzygium cumini* (L.) Skeels leaves were applied to sapwood blocks of *Pinus sylvestris* L. at 4000 mg/L^25^. Wood is a renewable resource that is vital to the global economy, yet insects and fungi that break down wood can attack it^26^. It is essential to develop low-impact, efficient wood protection solutions^27,28^. An alternative to employing synthetic or inorganic compounds as wood preservatives for wood protection is to use natural extractives from plants^29^. Globally, a great deal of study has been done on different plant and microorganism extracts. In order to prevent wood from biodegrading during production, storage, transit, and use, this study investigated the use of plant extracts for biological treatment.

Therefore, the current study aims to assess the bioactivity of extracts from *Nicotiana glauca* biomass (leaves and branches) to inhibit the fungal infestation or growth over the treated wood samples. The bioactive substances in the extracts were identified by chromatographic analyses using the GC-MS and HPLC tools.

## Materials and methods

### Preparation of the plant extracts

**Fig. 1:**
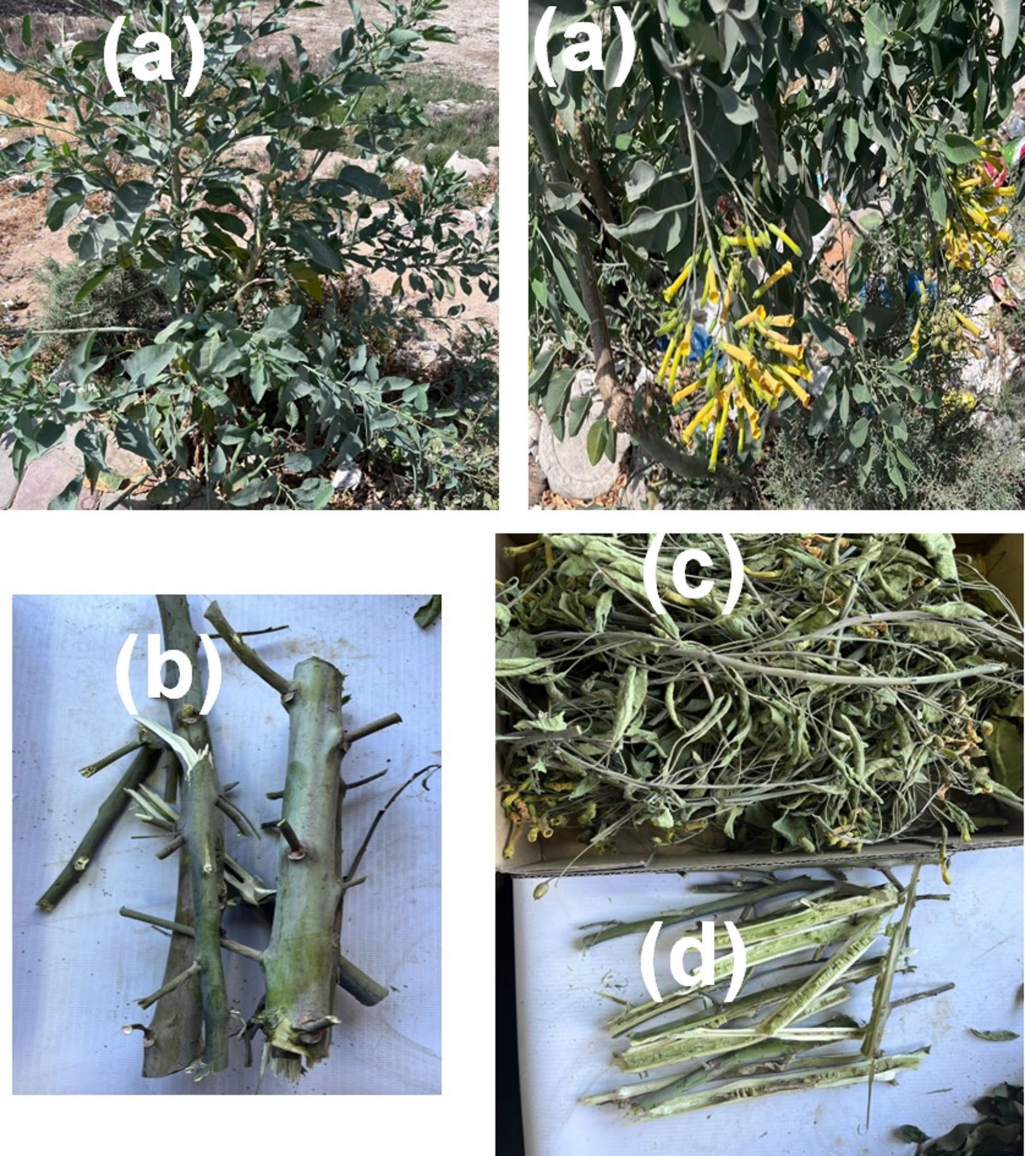
*Nicotiana glauca* biomass growth. (**a**) the whole plant showing the leaves and flowers; (**b**) branches; (**c**) dried plant with leaves; (**d**) dried branches. The height of the plant was measured to be around 1.5 m.

This study has complied with relevant institutional, national, and international guidelines and legislation. This study does not contain any studies with human participants or animals performed by any of the authors. *Nicotiana glauca* leaves and branches (Fig. 1) in the flowering stage were collected from the growing wild plants in Alexandria (Madrasa Al Qods Street, Coordinates 31.24977° N, 30.01987° E), Egypt, with permission from the Forestry and Wood Technology Department, Faculty of Agriculture (El-Shatby), Alexandria University, Alexandria, Egypt. The plant was identified under the voucher number ME890 by Dr. Mervat EL-Hefny (Department of Floriculture, Ornamental Horticulture and Garden Design, Faculty of Agriculture (El-Shatby), Alexandria University, Alexandria, Egypt. The plant was further identified and deposited at the Herbarium of the Plant Production Department, Faculty of Agriculture (Saba Basha), Alexandria University, Alexandria, Egypt.

After being cleaned with tap water to remove any dust or debris, the collected leaves and branches were allowed to air-dry at room temperature. A small laboratory mill was used to grind the dried leaves and branches into a powder. The extraction by the soaking method was used according to Salem et al.^30^ with some modification. Approximately 50 g of each powdered material (leaves and branches) was mixed with 200 mL of ethanol 70% and macerated by the soaking method for one week in a laboratory setting, with periodic hand-shaking, where every day it was agitated at least three times. This was to ensure a high amount of ethanol extracts (EEs) could be extracted from the powdered materials. The EEs of leaves and branches were then obtained by filtering the mixture through a cotton plug and Whatman filter paper No. 1. The EEs were vacuum-concentrated in a rotary evaporator. The concentrated extracts were placed in Petri dishes and allowed to air dry before further analysis. The yield of EEs was calculated from branches and leaves using the following formula^31^: Extract percentage = [extract amount (g)/air-dry sample amount (g)] × 100].

The percentages of EEs were 2.34% and 5.36%, from branches and leaves, respectively. Before further examination, the concentrated EEs were transferred into Petri dishes and allowed to air-dry^32^. The EEs were kept in sealed glass vials at 4 °C in a refrigerator^33^. The EEs were prepared at the concentrations of 1000, 500, 250, and 125 µg/mL by dissolving the respective amounts of extract in 10% dimethyl sulfoxide (10% DMSO).

## HPLC conditions for phytochemical analysis

An Agilent 1260 series instrument was used to perform the HPLC analysis of the EEs from *N. glauca* leaves and branches. A Zorbax Eclipse Plus C8 column (4.6 mm × 250 mm, id, 5 μm film thickness) was used for the separation. Water (A) and 0.05% trifluoroacetic acid in acetonitrile (B) at a flow rate of 0.9 mL/min made up the mobile phase. Using (A) concentrations of 82, 80, 60, 60, 82, 82, and 82%, respectively, a mobile phase linear gradient program was put into place with a step size of 1 min and periods of 5, 8, 12, 15, 16, and 20 min. The multi-wavelength detector was monitored at 280 nm. The injection volume was 5 µL for each sample solution (redissolved in acetone). The column temperature was maintained at 40 °C. Standard HPLC-grade phenolic and flavonoid compounds were used, including gallic acid, chlorogenic acid, catechin, methyl gallate, caffeic acid, syringic acid, pyrocatechol, rutin, ellagic acid, *p*-coumaric acid, vanillin, ferulic acid, naringenin, rosmarinic acid, daidzein, quercetin, cinnamic acid, kaempferol, and hesperetin. The identification of compounds was confirmed by comparing their retention times with those of the standard compounds. All chemical standards (high-performance liquid chromatography (HPLC grade) were from Sigma‒Aldrich (St. Louis, MO, USA)^34^. The HPLC chromatograms of the standard compounds are presented in our recent work^34^.

## GC-MS analysis of extracts

The chemical analysis of the EEs from *N. glauca* was performed using Gas chromatography-mass spectrometry (GC-MS). A Trace GC-TSQ mass spectrometer (Thermo Scientific, Austin, TX, USA) with a direct capillary column TG–5MS (30 m × 0.25 mm × 0.25 μm film thickness) was used^35,36^. The temperature of the column oven was first maintained at 50 °C, then raised by 5 °C per minute to 250 °C for two minutes, and finally raised by 30 °C per minute to 300 °C for two minutes. The MS transfer line and injector were maintained at 270 °C and 260 °C, respectively. As a carrier gas, helium was utilized at a steady flow rate of one milliliter per minute. The Autosampler AS1300, combined with GC in split mode, was used to automatically inject 1 µL diluted samples following a two-minute solvent delay. Using full scan mode, EI mass spectra were obtained at 70 eV ionization voltages in the m/z 50–650 range. The temperature of the ion source was adjusted to 200 °C. By comparing their mass spectra to those of the NIST 14 and WILEY 09 mass spectral databases, the components were identified^37^. The Xcalibur 3.0 data system and threshold settings of the GC-MS were used to confirm that all of the discovered compounds’ mass spectra were connected to the library. Additionally, the measurement match factor (MF) with values > 650 was used to confirm the identified chemicals^38^.

## Antifungal bioassay

The molecularly identified fungal isolates from the diseased root and branch samples from *Pinus halepensis* (Mill.): *Fusarium circinatum*, *Pythium tardicrescens*, and  *Phoma glomerata* (Accession numbers PV636492, PV636491, and PV892735), respectively^39^, were used for the antifungal activity. By referring to the NCBI and comparing the nucleotide sequences of the *Phoma* sp. isolate, we found that this is similar to *Phoma glomerata*.

Wood samples prepared at the dimension of 2 × 2 × 0.7 cm were obtained from *Fagus sylvatica* (L.) wood. Before conducting the fungal inoculation test, the generated wood samples were autoclaved at 121 °C for 20 min. The prepared ethanol extracts from *N. glauca* leaves and branches at the concentrations of 1000, 500, 250, and 125 µg/mL were applied to the wood samples. All the wood samples were subjected to antifungal evaluation against the growth of the three molds (*Phoma glomerata*, *F. circinatum*, and *P. tardicrescens*). Three replicates (wood samples) were used for each concentration and each isolated fungus. Each wood sample received 100 µL of the concentrated extract by soaking for 30 min.

Firstly, all the dishes were autoclaved at 121 °C for 20 min and left to cool, then a 15-day-old PDA culture of each fungus was prepared. The treated wood samples with the extracts, as well as the controls, were inoculated with each fungus disc (5 mm diameter) in a Petri dish that contained 15 mL of PDA culture and then incubated for 7 days at 25 ± 1 °C^39^. The fungal inhibition percentage (FIP) = [(Control growth - Growth in treatment)/Growth in control] × 100, for the treated and untreated woods against each fungus, was measured^32^. The positive control, viz., Cure-M 72% WP (Mancozeb 64%+Metalaxyl 8%), was tested at the recommended dosage (2000 µg/mL) for antifungal activity by the poisoned food technique^40^. 10% DMSO was used as a negative control sample. The minimum inhibitory concentrations (MICs) of the ethanol extracts from *N. glauca* leaves and branches prepared at concentrations from 15.6 to 250 µg/mL were assessed using the broth dilution method according to CLSI^41^.

### Statistical analysis

The percentage of fungal inhibition measured from the treated wood with the concentrated extracts (1000, 500, 250, and 125 µg/mL) from leaves and branches was statistically analyzed using two-way ANOVA (analysis of variance) in SAS software (SAS Institute, Release 8.02, Cary, North Carolina, USA). The means from each treatment of the extracts and their concentrations were compared to the positive and negative control treatments using Duncan’s Multiple Range Test.

## Results

### Phenolic and flavonoid compounds by HPLC

**Fig. 2:**
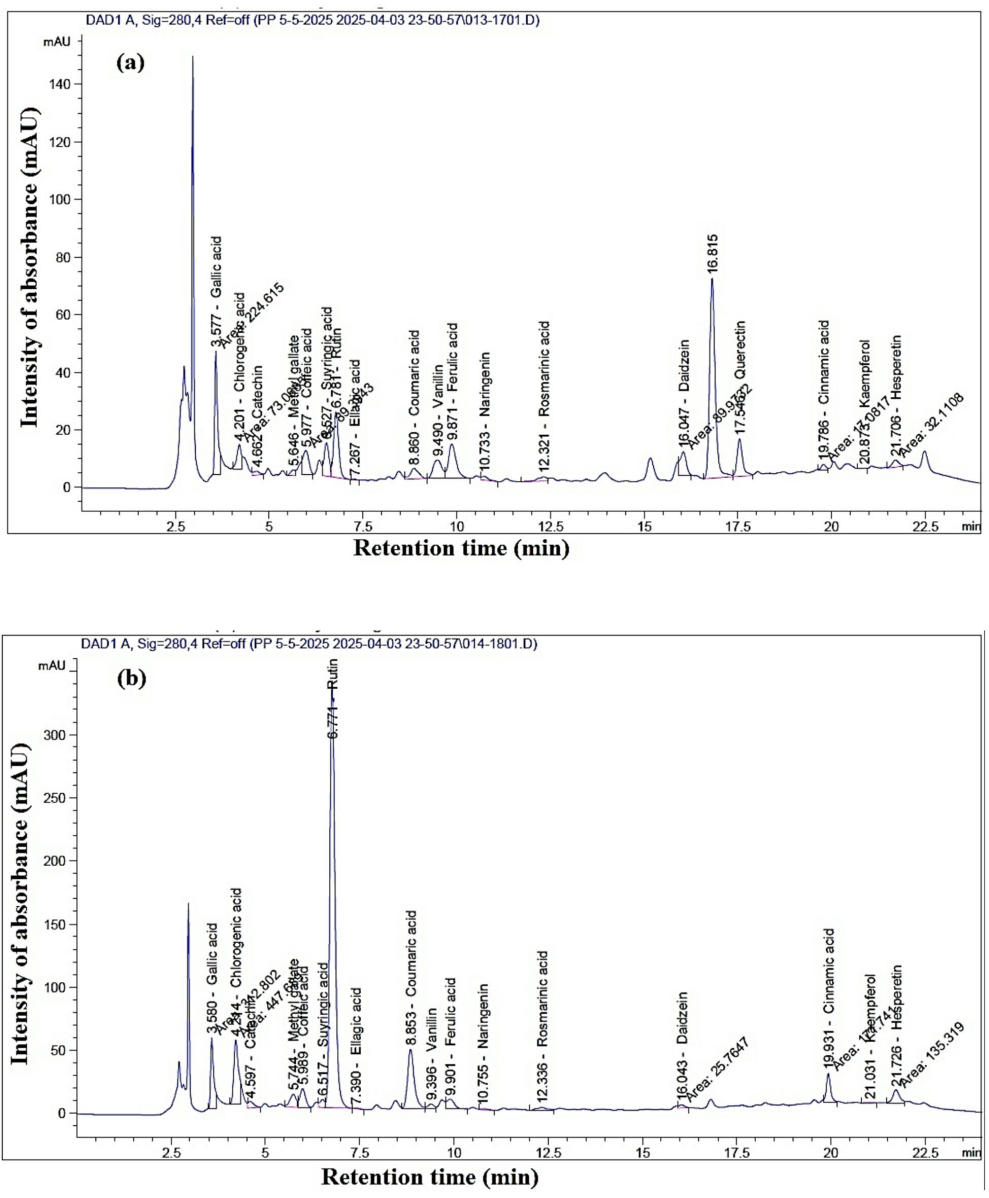
The phenolic and flavonoid compounds identified in the ethanol extracts from *Nicotiana glauca* by HPLC analysis. (**a**) branches (**b**) leaves.

Table 1; Fig. 2a show the chemical compounds identified in the ethanol extract (EE) from branches of *Nicotiana glauca*. The abundant phenolic and flavonoid compounds in the EE by HPLC from the branch extract were rutin (1529.37 µg/g dry extract), quercetin (856.96 µg/g dry extract), gallic acid (813.79 µg/g dry extract), chlorogenic acid (511.95 µg/g), ferulic acid (498.86 µg/g dry extract), syringic acid (339.03 µg/g dry extract), caffeic acid (267.57 µg/g dry extract), and daidzein (257.22 µg/g dry extract).

**Table 1:** Phenolic and flavonoid compounds identified in the ethanol extract of *Nicotiana glauca* branches.

Retention time (min)	Compound	Area (mAU*s)	Conc. (µg/g dry extract)
3.577	Gallic acid	224.615	813.79
4.201	Chlorogenic acid	73.068	511.95
4.662	Catechin	15.093	175.21
5.646	Methyl gallate	15.501	43.94
5.977	Caffeic acid	89.394	267.57
6.527	Syringic acid	105.916	339.03
6.781	Rutin	210.384	1529.37
7.267	Ellagic acid	1.677	9.53
8.860	Coumaric acid	58.137	106.15
9.490	Vanillin	104.028	176.05
9.871	Ferulic acid	172.513	498.86
10.733	Naringenin	16.683	80.65
12.321	Rosmarinic acid	24.110	107.11
16.047	Daidzein	89.973	257.22
17.546	Quercetin	132.347	856.96
19.786	Cinnamic acid	17.081	18.49
20.873	Kaempferol	1.047	5.37
21.706	Hesperetin	32.110	75.92

Table 2; Fig. 2b show the chemical compounds identified in the EE from leaves of *N. glauca*. The most abundant compounds were rutin (23364.18 µg/g dry extract), chlorogenic acid (3136.67 µg/g dry extract), gallic acid (1133.30 µg/g dry extract), coumaric acid (1066.13 µg/g dry extract), catechin (647.99 µg/g dry extract), caffeic acid (447.27 µg/g dry extract), methyl gallate (337.14 µg/g dry extract), ferulic acid (336.49 µg/g dry extract), and hesperetin (319.94 µg/g dry extract).

**Table 2:** Phenolic and flavonoid compounds identified in the ethanol extract from *Nicotiana glauca* leaves.

Retention time (min)	Compound	Area (mAU*s)	Conc. (µg/g dry extract)
3.580	Gallic acid	312.802	1133.30
4.214	Chlorogenic acid	447.688	3136.67
4.597	Catechin	55.818	647.99
5.744	Methyl gallate	118.939	337.14
5.989	Caffeic acid	149.429	447.27
6.517	Syringic acid	54.266	173.70
6.771	Rutin	3214.045	23364.18
7.390	Ellagic acid	0.61	30.44
8.853	Coumaric acid	21.32	1066.13
9.396	Vanillin	1.64	81.91
9.901	Ferulic acid	6.73	336.49
10.755	Naringenin	1.08	53.95
12.336	Rosmarinic acid	4.06	203.12
16.043	Daidzein	1.47	73.66
19.931	Cinnamic acid	3.72	185.86
21.031	Kaempferol	0.70	34.95
21.726	Hesperetin	6.40	319.94

### GC-MS of the ethanol extracts

**Fig. 3:**
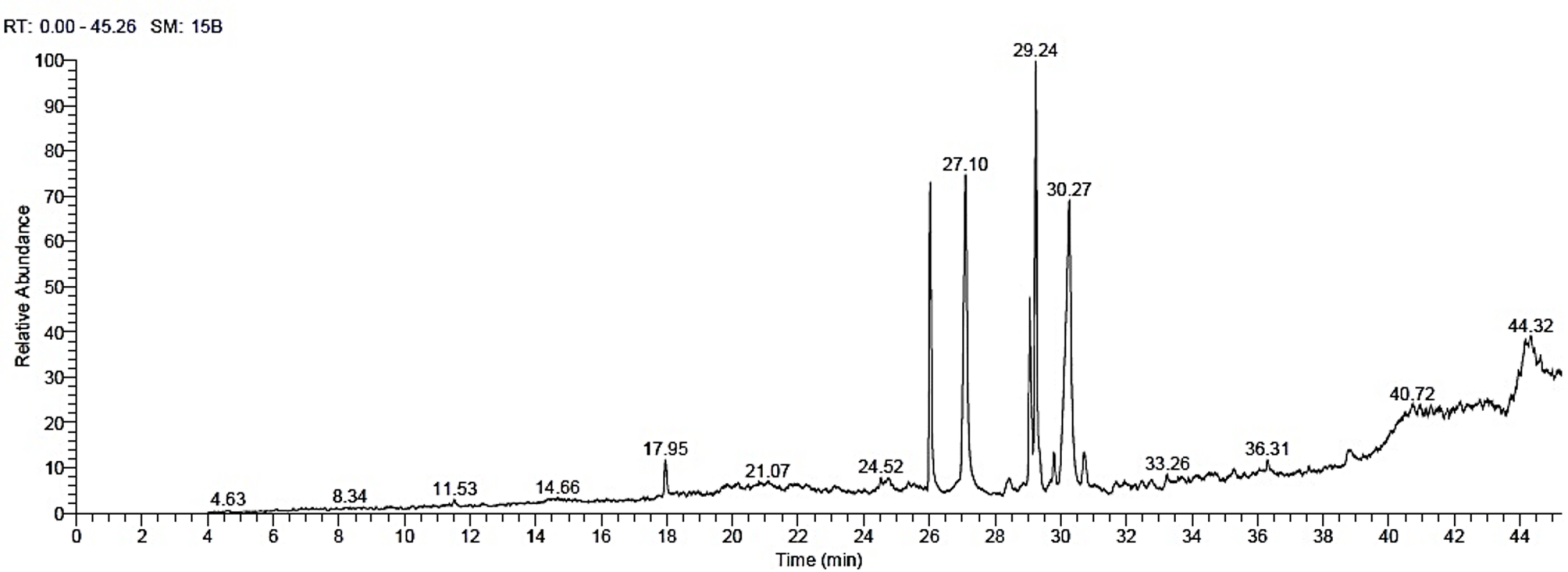
The GC-MS chromatograms of the identified compounds in the ethanol extract from *Nicotiana glauca* branches.

The chemical compounds identified in the EE of *N. glauca* branches are shown in Table S1 and Fig. 3. The main compounds were presented in Table 3 as methyl oleate (19.39%), oleic acid (17.09%), 9-octadecenal (15.65%), methyl palmitate (14.08%), and methyl 12,13-tetradecadienoate (8.99%), which were the most abundant compounds.

**Table 3:** The main chemical compounds by GC-MS from the ethanol extract of *Nicotiana glauca* branches.

RT	Area %	Compound	Match factor	Molecular formula
26.03	14.08	Methyl palmitate	919	C_17_H_34_O_2_
27.11	17.09	Oleic acid	837	C_18_H_34_O_2_
28.43	1.42	Benzyl (6Z,9Z,12Z)−6,9,12-octadecatrienoate	745	C_25_H_36_O_2_
29.06	8.99	Methyl 12,13-tetradecadienoate	824	C_15_H_26_O_2_
29.24	19.39	Methyl oleate	863	C_19_H_36_O_2_
29.80	1.86	Methyl dihydrohydnocarpate	766	C_12_H_22_O_2_
30.27	15.65	9-Octadecenal	849	C_18_H_34_O
30.71	2.23	Hydnocarpic acid	772	C_16_H_28_O_2_
31.70	1.08	N-[5-hydroxy-n-pentyl]-Arachidonic amide	705	C_25_H_43_NO_2_
32.46	1.59	2,3-Bis(acetyloxy)propyl (9E,12E,15E)−9,12,15-octadecatrienoate	673	C_25_H_40_O_6_
38.76	1.92	12-Methyl-E, E-2,13-octadecadien-1- ol	686	C_19_H_36_O

**Fig. 4:**
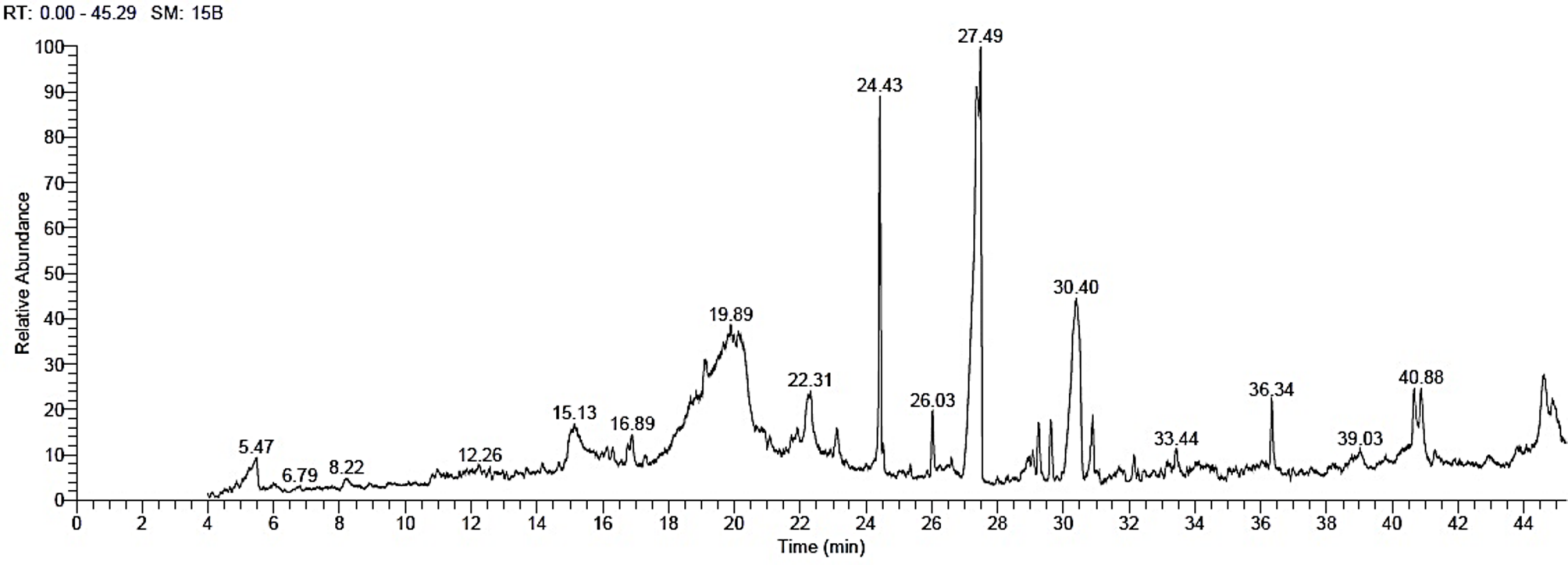
The GC-MS chromatograms of the identified compounds in the ethanol leaf extract from *Nicotiana glauca*.

The chemical compounds identified in the EE from *N. glauca* leaves are shown in Table S2 and Fig. 4. The main compounds in Table 4 were anabasine (11.44%), palmitic acid (11.29%), oleic acid (10.96%), hydnocarpic acid (8.34%), hexahydrofarnesyl acetone (6.76%), phytosphingosine (5.63%), tert-hexadecanethiol (4.31%), atrazine deisopropyl (3.74%), and tetrahydrocannabihexol (2.03%).

**Table 4:** The GC-MS analysis of the chemical compounds from the ethanol extract of *Nicotiana glauca* leaves.

RT	Area %	Compound	Match factor	Molecular formula
5.48	1.41	3-Methylvaleric acid	679	C_6_H_12_O_2_
15.03	1.33	5-Hydroxy-4-hydroxymethyl-1-(1-hydroxy-1-isopropyl)cyclohex-3-ene	682	C_10_H_18_O_3_
19.11	1.19	Methyl 8,11-octadecadiynoate	703	C_19_H_30_O_2_
19.89	11.44	Anabasine	708	C_10_H_14_N_2_
22.24	1.36	1-Cyclohexene-1-methanol	728	C_7_H_12_O
22.32	1.54	3-Oxiranyl-7-oxabicyclo[4.1.0]heptane	784	C_8_H_12_O_2_
23.12	1.56	5α-Androstan-16-one, cyclic ethylene mercaptole	709	C_21_H_34_S_2_
24.43	6.76	Hexahydrofarnesyl acetone	784	C_18_H_36_O
26.02	1.30	Cyclopentaneundecanoic acid methyl ester	771	C_17_H_32_O_2_
27.36	10.96	Oleic acid	774	C_18_H_34_O_2_
27.49	11.29	Palmitic acid	772	C_16_H_32_O_2_
28.90	1.05	1-Hexadecanol, acrylate	810	C_19_H_36_O_2_
29.24	1.49	7-Nonenoic acid methyl ester	784	C_10_H_18_O_2_
29.63	1.39	Isophytol	713	C_20_H_40_O
30.38	8.34	Hydnocarpic acid	830	C_16_H_28_O_2_
30.42	5.63	Phytosphingosine	819	C_18_H_39_NO_3_
30.90	1.98	Dodecanoic acid	705	C_12_H_24_O_2_
40.87	4.31	*tert*-Hexadecanethiol	651	C_16_H_34_S
44.62	3.74	Atrazine deisopropyl	705	C_5_H_8_ClN_5_
44.85	1.98	4-t-Butyl-2-(1-methyl-2-nitroethyl)c yclohexanone	709	C_13_H_23_NO_3_

### Antifungal activity

**Fig. 5:**
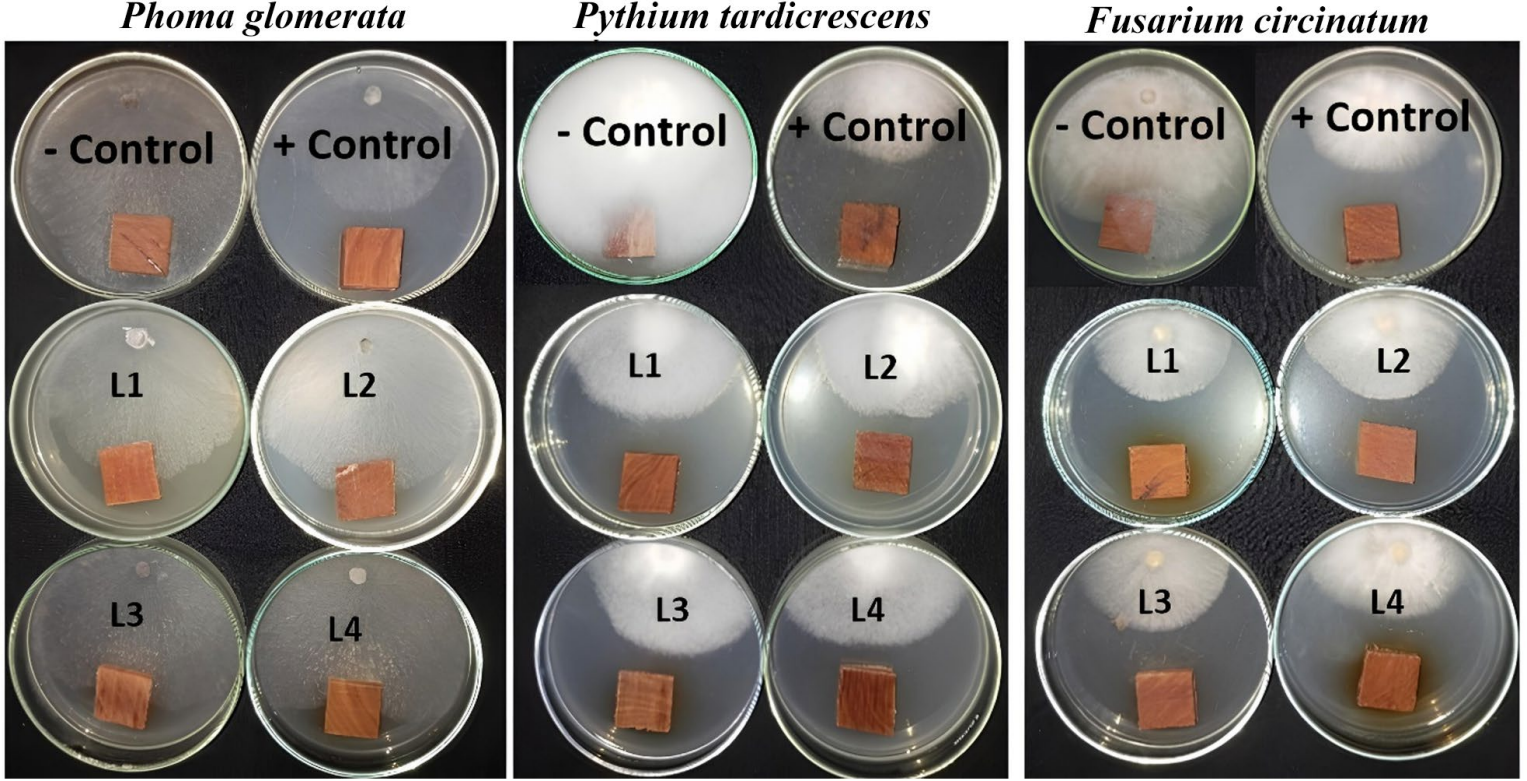
Visual observation of the antifungal activity of the branch ethanol extract when applied to *Fagus sylvatica* wood samples against the growth of *Pythium tardicrescens*, *Fusarium circinatum*, and *Phoma* *glomerata*. The concentrations of the branch extract are labeled as L1, L2, L3, and L4 (1000, 500, 250, and 125 µg/mL), respectively. +Control (positive control): Cure-M 72% WP (Mancozeb 64%+Metalaxyl 8%); -Control (negative control): 10% DMSO.

**Fig. 6:**
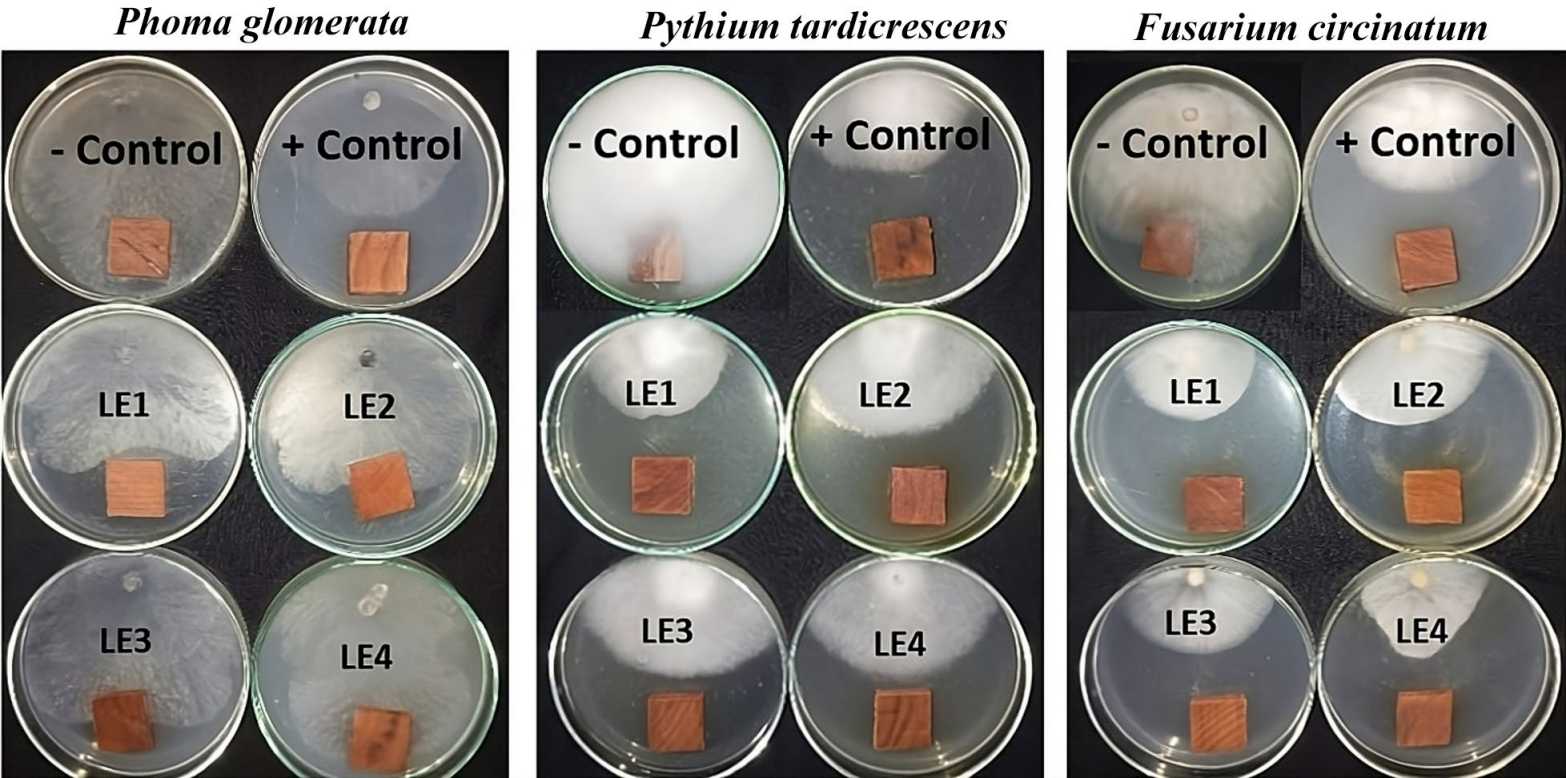
Visual observation of the antifungal activity of the leaf ethanol extract when applied to *Fagus sylvatica* wood samples against the growth of *Pythium tardicrescens*, *Fusarium circinatum*, and *Phoma glomerata*. The concentrations of the leaf extract are labeled as LE1, LE2, LE3, and LE4 (1000, 500, 250, and 125 µg/mL), respectively. +Control (positive control): Cure-M 72% WP (Mancozeb 64%+Metalaxyl 8%); -Control (negative control): 10% DMSO.

Table 5 presents the antifungal activity of the EEs from *N. glauca* branches and leaves against the growth of *Phoma* *glomerata*, *Pythium tardicrescens*, and *Fusarium circinatum*. Figures 5 and 6 show the visual observation of the incubated fungi with the treated *Fagus sylvatica* wood samples by the EEs from *N. glauca* branches and leaves, respectively.

Leaf and branch EEs at 1000 µg/mL exhibited the highest activity against the growth of *P. glomerata*, with moderate fungal inhibition percentage (FIP) values of 35.92% and 27.77%, respectively, compared to the positive control Cure-M 72% WP (FIP 36.29%). At the concentration of 1000 µg/mL, the leaf and branch EEs observed the highest FIP values of 58.15% and 47.41%, respectively, against the growth of *Pythium tardicrescens* compared to the positive control Cure-M 72% WP (FIP 55.18%). The highest activity of branch and leaf EEs against *Fusarium circinatum* was observed at the concentration of 1000 µg/mL, with FIP values of 55.55 and 55.18%, respectively, compared to the positive control Cure-M 72% WP (FIP 48.15%).

The EE from the branches had MIC values of 31.25, 62.5, and 125 µg/mL, whereas the EE from the leaves had MIC values of 15.25, 62.6, and 250 µg/mL against the growth of *Fusarium circinatum*, *Pythium tardicrescens*, and *P. glomerata*, respectively.

**Table 5:** The fungal growth inhibition (%) of the ethanol extracts from branches and leaves of *Nicotiana glauca* against the growth of *Pythium tardicrescens*, *Fusarium circinatum*, and *Phoma* *glomerata* when applied to *Fagus sylvatica* wood.

Treatment	Concnetration	Fungal growth inhibition (%)		
		*Phoma* *glomerata*	*Pythium* *tardicrescens*	*Fusarium* *circinatum*
NC	10% DMSO	0.00 F	0.00E	0.00E
PC	Cure-M 72% WP	36.29 A ± 2.79	55.18 A ± 0.64	48.15D ± 1.28
Branch extract	1000 µg/mL	27.77B ± 5.55	47.41B ± 1.28	55.55 A ± 1.11
	500 µg/mL	17.03D ± 1.69	45.92B ± 0.64	50.74 C ± 1.28
	250 µg/mL	10.00E ± 4.006	43.70BC ± 1.69	48.52D ± 0.64
	125 µg/mL	4.44 F ± 3.33	41.48 C ± 0.64	47.41D ± 0.64
	MIC µg/mL	125	62.5	31.25
Leaf extract	1000 µg/mL	35.92 A ± 2.56	58.15 A ± 6.51	55.18 A ± 0.64
	500 µg/mL	22.22 C ± 1.11	46.66B ± 1.11	53.33B ± 0.00
	250 µg/mL	15.18D ± 1.69	41.85 C ± 0.64	52.22B ± 0.00
	125 µg/mL	2.59 F ± 1.69	35.55d ± 1.11	47.41D ± 1.28
	MIC µg/mL	250	62.5	15.6
P-value		0.0272	< 0.0001	0.0011

Values are means ± SD; Means with the same letter are not significantly different according to Duncan’s Multiple Range Test. PC (Positive control): Cure-M 72% WP (Mancozeb 64%+Metalaxyl 8%); NC (Negative control): 10% DMSO.

## Discussion

*Nicotiana glauca* contains a variety of phenolic and flavonoid compounds, which vary in concentration depending on the extraction method and the part of the plant used. These compounds contribute to the plant’s antioxidant and antimicrobial properties. Several phenolic and flavonoid compounds like gallic acid, chlorogenic acid, catechin, methyl gallate, caffeic acid, syringic acid, rutin, ellagic acid, coumaric acid, vanillin, ferulic acid, naringenin, rosmarinic acid, daidzein, quercetin, cinnamic acid, kaempferol, and hesperetin were isolated and identified from the ethanol extracts (EEs) of branches and leaves of *N. glauca*. Except that quercetin was not detected in the leaf EE. These EEs showed different activities when applied to *Fagus sylvatica* wood samples against the growth of *Pythium tardicrescens*, *Fusarium circinatum*, and *Phoma* *glomerata*.

The EE from the branch showed the abundant phenolic and flavonoid compounds rutin, quercetin, gallic acid, chlorogenic acid, ferulic acid, syringic acid, caffeic acid, and daidzein. While in the leaf EE, the abundant compounds were rutin, chlorogenic acid, gallic acid, coumaric acid, catechin, caffeic acid, methyl gallate, ferulic acid, and hesperetin.

Saponins, coumarins, tannins, phlobatannins, resins, cardiac glycosides, and steroids were found in the stem and leaves of *N. glauca*, as well as terpenoids in the root, according to the initial phytochemical screening. Flavonoids were found in every part of the plant^13^. Chlorogenic acid, rutin, hyperoside, robinetin, umbelliferone (coumarin derivative), scopoletin (coumarin derivative), saponarin, and syringic acid were all detected in the leaf extract^8^. Quercetin and kaempferol, as well as several of their glycosides (rutin, kaempferol-3-glucoside, and quercetin-3-glucoside), cinnamic, and ferulic acids, were detected by flavonols in the leaf extract^13^. According to another study, *N. glauca* leaves have higher levels of quercetin, cinnamic acid, and rutin than various vegetables, which is consistent with flavonoid concentrations^42^.

According to the chemical compounds’ GC-MS analysis, some carboxylic acids and esters, saturated fatty acids, and hydrocarbon compounds were found. There were also some alkaloids found. Methyl oleate, oleic acid, 9-octadecenal, methyl palmitate, and methyl 12,13-tetradecadienoate were found in significant amounts in the branch extract, whereas the leaf extract contained anabasine, palmitic acid, oleic acid, hydnocarpic acid, hexahydrofarnesyl acetone, phytosphingosine, tert-hexadecanethiol, and atrazine deisopropyl.

The methanol extract from leaves showed the presence of ethylene oxide, acetaldehyde, dimethyl sulfide, pentanal, 1-propanal, and propanal, 2-methyl as the most active components in^19^. The volatile oil of the *N. glauca* extract was found to include significant amounts of oxygenated sesquiterpenes, including β-bisabolol, carboxylic acids, and esters such as ethyl linoleate and hexadecanoic acid. Additionally, 9,17-octadecadienal was found^43^. The predominant ingredient in *N. glauca* leaves extract was eugenol, followed by nonadecane, eugenyl acetate, 3-methyl-tridecane, and 8-methyl-heptadecane^44^. In another investigation, the stem of *N. glauca* active fraction contained a variety of polyphenols and aromatic compounds, such as bicyclo heptanes, 3, 7, 11, 15-tetramethyl-2-hexadecen-1-ol, scopoletin, D-α-tocopherol, campesterol, stigmasterol, and β-sitosterol^45^.

Hexacosenol, nonacosane, triacontane, octacosenol, nonacosonal, hentriacontane, dotriacontane, tritriacontane, and total hydrocarbon were the nine components found in the hexane extract of *N. glauca* leaves^46^. *N. glauca* leaf extracts from seven different places revealed that the most prevalent chemicals were 2-acetoxyisobutyryl chloride, 2,4-dimethyl-1,3-dioxolane-2-methanol, and 4-hydroxy-4-methyl-2-pentanone^47^. Anabasine, a pyridine and piperidine alkaloid, was discovered to be present in varying levels in all places under investigation, particularly in the methanol extracts^47^. Compounds like 2-methyl-6-pheny-l1,6-heptadiene, eicosane, octadecenoic acid methyl ester, dodecanoic acid 1-methylethyl ester, diethyl phthalate, (*z*,*z*,*z*)−9,12,15-octadecatrienoic acid, 1 H-2,3-dihydro-1,1,3-trimethyl-3-phenyl-Indene, 11,14,17-eicosatrienoic acid methyl ester, phytol, 15-methyl-hexadecanoic acid methyl ester, 2-hydroxy-propanoic acid ethyl ester, and tetratetracontane were also reported in the ethanol extracts from *N. glauca* leaves as collected from various locations in Saudi Arabia^47^. The essential oil from *N. glauca* was found to contain a high percentage of saturated hydrocarbons, with hexadecane, limonene, and heneicosane being the minor ingredients (about 0.5% each)^44^.

Anabasine, anabaseine, nicotine, nornicotine, ammodendrine, rutin, and chlorogenic acid were found in the alkaloid-rich fraction analyzed by the UPLC-HRESI-MS in *N. glauca*. However, the GC-MS identified 5-pentanolactam, anabasine, 1,2,3,4-tetrahydro-pyridine-2,5-dicarbonitrile, 1,2,3,5,9b-pentaaza-cyclopenta[a]naphthalen-4-ol, and 1-ethynyl-1-isocyano-cyclohexane as the main constituents^43^.

According to the antifungal activity of the EEs from branches and leaves of *N. glauca*, the fungal growth of *Phoma* *glomerata*, *Fusarium circinatum*, and *Pythium tardicrescens* was inhibited by the application of the EE from the leaves on *Fagus sylvatica* wood samples more than using the branch EE. These could be related to the presence of bioactive compounds as determined by HPLC and GC-MS.

For the antifungal activity of *N. glauca* extracts, the leaf and flower extracts at the tested concentrations 1, 2, 3, and 4% showed potential effects against the growth of *Trichoderma viride* and *Fusarium oxysporum* f. sp. *melonis*^48^. However, *F. oxysporum* f. sp. *lycopersici* and *F. oxysporum* f. sp. *tuberosi* were found to be less sensitive to *N. glauca* extracts as compared to *F. oxysporum* f. sp. *melonis*. At the concentrations of 1, 2, and 3%, the aqueous extract from *N. glauca* leaves was observed to have the strongest antifungal efficacy against *Aspergillus niger*, *A. flavus*, *A. terreus*, *Alternaria alternata*, and *Rhizoctonia solani* among a few medicinal plants^49^. In terms of the measured MIC values, the acetone extract from *N. glauca* leaves demonstrated good antifungal activity against the growth of *A. niger*, *A. parasiticus*, *Colletotrichum gloeosporioides*, *F. oxysporum*, *T. harzianum*, *Phytophthora nicotiana*, *P. ultimum*, *R. solani*, and *Penicillium janthinellum*^21^.The methanol extracts of *Ceratonia siliqua* L. leaves and branches exhibited the strongest action against *A. alternata* growth at 4%, whereas the leaf extract demonstrated the strongest fungal inhibition against *F. oxysporum*^50^. These could be related to the presence of phenolic and flavonoid compounds like catechin, syringic acid, gallic acid, coumaric acid, and methyl gallate in the extract^50^. The highest inhibition of fungal growth against *Rhizoctonia solani* was observed by the application of methanol extracts from *Ziziphus spina-christi* (L.) Desf. branches and leaves on *Pinus sylvestris* wood blocks^50^. These also could result from the presence of polyphenolic compounds such as ellagic acid, gallic acid, rutin, catechin, and chlorogenic acid^50^. Ethanol extractives from teak heartwood (*Tectona grandis* L.f.) waste significantly changed the decay resistance of the treated wood from 10-year-old teak sapwood and *Pinus* sp. against the brown rot fungus *Postia placenta*^51^. Wood samples from *Vitex doniana* Sweet, and *Triplochiton scleroxylon* K.Schum. treated with *Lawsonia inermis* L. extracts from the bark and leaves exhibited good activity against the growth *of Ganoderma lucidum* and *Sclerotium rolfsii* in comparison to the untreated wood samples^52^.

The formulation of environmentally friendly wood preservatives may benefit from the antifungal properties of phenolic chemicals. When compared to the untreated control specimens, Scots pine and beech wood specimens impregnated with leaf extracts of Sicilian sumac (*Rhus coriaria* L.), valonia oak (*Quercus macrolepis* L.), and Turkish pine bark (*Pinus brutia* Ten.) demonstrated enhanced resistance to *Trametes versicolor* (beech wood) and *Gloeophyllum trabeum* (pine wood)^53^. Galangin and pinocembrin, two flavonoids, showed similar antifungal effectiveness to ketoconazole against *Ganoderma applanatum*, *Pycnoporus sanguineus*, *Schizophyllum commune*, and *Aspergillus niger*^54^. The antifungal ability of flavonoids extracted from *Citrus* species, including hesperidin, naringenin, and neohesperidin, as well as their enzymatically modified derivatives, was observed against the growth *of Aspergillus parasiticus*,* Aspergillus flavus*,* Fusarium semitectum*, and *Penicillium expansum*^55^.

The extracts from *N. glauca* presented some bioactive phenolic and flavonoid compounds. Many flavonoids are susceptible to degradation by environmental factors, such as sunlight^56^. Rutin is a flavonoid found in many plants that has shown promising antifungal activity in laboratory and animal studies, particularly against *Candida* and *Aspergillus* fungal species^57^. Rutin has demonstrated antifungal properties, including inhibiting the growth of *Candida albicans* and *Cryptococcus neoformans*^58^. Although rutin has shown encouraging antifungal properties against *Gloeophyllum trabeum* and *Trametes versicolor* in a lab setting, there is no proof that it is marketed as a stand-alone wood protection product^59^. Rutin is a bioactive plant molecule that may be able to protect wood, but its practical use is limited by its weak water solubility and low absorption. This would need to be addressed using specialized formulation processes to create a viable product^60^.

Quercetin is one of the flavonoids being studied by researchers as a possible ingredient in environmentally friendly wood treatments^61^. The majority of studies on quercetin’s antifungal properties concentrate on laboratory and medical usage rather than wood preservation. The research that deals with wood is still in its early stages. The stability and bioavailability of quercetin as a preservative are unknown, and although it has shown antifungal properties in lab conditions against certain fungi, such as *Aspergillus* and *Candida*, its direct application as a stand-alone antifungal wood treatment is still in the experimental research stage.

Although gallic acid, like quercetin, has shown antifungal properties in experimental conditions, it is not yet marketed as a stand-alone wood treatment. It is a polyphenol and a part of hydrolyzable tannins, which are found naturally in some plants and help prevent wood from decaying. Its application in environmentally friendly wood preservatives is still being studied; it is frequently mixed with other materials to increase its efficacy and fixation^62^. The concentration, the kind of wood, and the particular fungus species can all have a substantial impact on the antifungal effectiveness of plant extracts containing gallic acid^63^. A mixture of citric acid and tannin-rich inner and outer bark extracts from sugar maple (*Acer saccharum* Marshall), when applied to *Leucaena leucocephala* (Lam.) de Wit wood, was found to have a strong anti-mold efficacy. Salicylic acid, gallic acid, and *p*-hydroxybenzoic acid were identified as the primary constituents of biological activity^64^. High antifungal activity against *F. solani* is exhibited by purified gallic acid, and this activity was dose-dependent. The hyphae shrank and collapsed after being incubated with gallic acid (500 ppm) for 24 h^65^. Gallic, protocatechuic, vanillic, chlorogenic, caffeic, and ferulic acids from tobacco waste were observed as antifungal agents against wood-decay fungi^66^.

Like quercetin and gallic acid, chlorogenic acid is a polyphenol that has antifungal properties in lab settings but is not used as a stand-alone commercial wood preservative. Chlorogenic acid is being investigated as a possible natural and environmentally friendly wood preservative, particularly when combined with other compounds or as a component of complex plant extracts^63^. Extracts from *Schotia brachypetala* branches containing chlorogenic and gallic acids as main compounds were effective in inhibiting the growth of certain pathogenic fungi on wood samples^34^. Chlorogenic acid extracted from coffee grounds can inhibit wood-decaying fungi in a lab setting, suggesting potential for developing a “green” preservative from organic waste^67^.

Although the polyphenol ferulic acid has shown antifungal qualities in experimental conditions, it is not yet a widely available, stand-alone wood treatment. Ferulic acid is being investigated as a potential ingredient in the ongoing search for natural and environmentally friendly alternatives to traditional wood preservatives. But like other natural substances, it has drawbacks, such as being prone to leaching^68^. Ferulic acid can inhibit the growth of various fungi, including some wood-decaying types. A study found that ferulic acid inhibited the activity of 26 S fungal proteasomes, which are crucial for fungal cellular function^69,70^.

Phenolic compounds combat fungi through a variety of, frequently combined, mechanisms, including disrupting cell membranes (increasing permeability, altering integrity)^71,72^. Additionally, they produce reactive oxygen species (ROS) for oxidative stress, interfering with essential enzymes like those in ergosterol synthesis, preventing spore germination, influencing adhesion/biofilm formation, and affecting important fungal pathways like Ras/cAMP^73–75^. These mechanisms ultimately result in cell death or growth inhibition. Certain phenolics, such as ellagic acid and caffeic acid phenethyl ester (CAPE), interfere with cell wall integrity, potentially by inhibiting 1,3-β-glucan synthase, an enzyme critical for cell wall synthesis^76^.

It is essential to remember that although *N. glauca* contains some beneficial substances, its high concentration of alkaloids, such as nicotine and anabasine, makes it extremely hazardous. Ingestion of this toxin can be lethal, making it a serious health risk^14^. Therefore, the safety considerations and implications for practical use as a wood preservative are required for further research.

The differences in antifungal effectiveness between *Nicotiana glauca* plant sections and concentrations imply that greater research into these extracts may result in wood preservative compositions that are more successful. Furthermore, *Nicotiana glauca* is a useful source of bioactive chemicals due to its potential to inhibit the growth of molds. However, before the obtained extracts can be utilized as alternatives to synthetic fungicides, more research is required to evaluate the toxicity and effectiveness of the treatments for long-term usage as antifungal agents.

It is crucial to remember that bioactivity data collected in a lab setting cannot always correspond to in vivo toxicity. Therefore, this work paves the way for more investigation into the long-term effects, or shelf life, of *Nicotiana glauca* extracts when applied as a wood-biofungicide.

## Conclusions

For new and significant uses of natural products of *Nicotiana glauca* extracts, the ethanol extracts from leaves and branches were applied to wood samples. They observed good inhibition of the growth of fungi including *Phoma* *glomerata*, *Fusarium circinatum*, and *Pythium tardicrescens*. The extracts were found to have several bioactive compounds as analyzed by HPLC and GC-MS apparatuses. These compounds are listed as phenolic and flavonoids, as well as several hydrocarbons, saturated fatty acids, and alkaloid compounds. *Nicotiana glauca* could be considered a valuable natural resource of both flavonoids and phenolic compounds with good wood-biofungicide activities.

## Supplementary Information

Below is the link to the electronic supplementary material.

Supplementary Material 1

## Data Availability

All data generated or analyzed during this study are included in this published article.
