# Supplementary material for: Unveiling the antifungal potential of extracts in leaves and branches from Nicotiana glauca for wood biofungicides
Source: Sci Rep. 2026 Mar 27;16:10822. doi: 10.1038/s41598-026-42531-x (PMC13039992; doi:10.1038/s41598-026-42531-x)
Supplement: Supplementary file 1 — Supplementary Material 1 [file 41598_2026_42531_MOESM1_ESM.docx]

Unveiling the antifungal potential of extracts from leaves and branches of *Nicotiana glauca*

Mohamed Z. M. Salem^a^, Abeer A. Mohamed^b^, Mohammed A. A. Elshaer^c^, Mohamed A. M. Abd-Elraheem^c^, Zakaria H. Saad^d^, Maisa M. A. Mansour^e^, Mervat EL-Hefny^f,^*

*^a^ Forestry and Wood Technology Department, Faculty of Agriculture (El-Shatby), Alexandria University, Alexandria 21545, Egypt;* [mohamed-salem@alexu.edu.eg](mailto:mohamed-salem@alexu.edu.eg)

*^b^ Plant Pathology Research Institute, Agriculture Research Center (ARC), Alexandria 21616, Egypt;* [abeera.mohamed81@gmail.com](mailto:abeera.mohamed81@gmail.com)

*^c^ Agricultural Biochemistry Department, Faculty of Agriculture, Al-Azhar University, Sadat, Egypt;* [*mmm_elshaer@azhar.edu.eg*](mailto:mmm_elshaer@azhar.edu.eg)*;* [*mohamedawad@azhar.edu.eg*](mailto:mohamedawad@azhar.edu.eg)

*^d^ Agricultural Biochemistry Department, Faculty of Agriculture, Al-Azhar University, Cairo, Egypt;* [*Zakaria.Hassan@azhar.edu.eg*](mailto:Zakaria.Hassan@azhar.edu.eg)

*e Organic Materials Conservation Department, Faculty of Archaeology, Cairo University, 12613 Giza, Egypt;* [*maisamansour@cu.edu.eg*](mailto:maisamansour@cu.edu.eg)

*^f^ Department of Floriculture, Ornamental Horticulture and Garden Design, Faculty of Agriculture (El-Shatby), Alexandria University, Alexandria 21545, Egypt;* [mervat.mohamed@alexu.edu.eg](mailto:mervat.mohamed@alexu.edu.eg)

*: Corresponding author: [mervat.mohamed@alexu.edu.eg](mailto:mervat.mohamed@alexu.edu.eg)

Table S1. Chemical compounds by GC-MS from the ethanol extract of *Nicotiana glauca* branches

| RT | Area % | Compound | Match factor | Molecular Formula |
| --- | --- | --- | --- | --- |
| 11.52 | 0.33 | 1,9-Decadiyne | 745 | C_10_H_14_ |
| 17.95 | 1.94 | 2-Hydroxy-1,4,4-trimethyl-  bicyclo[3.1.0]hexane-6-methanol | 699 | C_10_H_18_O_2_ |
| 18.31 | 0.19 | 3-Chloropropionic acid, undec-2-enyl ester | 636 | C_14_H_25_ClO_2_ |
| 18.68 | 0.27 | *cis*-4-Decenal | 737 | C_10_H_18_O |
| 18.95 | 0.25 | 17à-Hydroxy-17á-cyano-preg-4-en-3-one | 640 | C_20_H_27_NO_2_ |
| 19.86 | 0.26 | Desulphosinigrin | 655 | C_10_H_17_NO_6_S |
| 20.11 | 0.16 | 2-Aminoethanethiolsulfuric acid | 672 | C_2_H_7_NO_3_S_2_ |
| 20.20 | 0.28 | d-Mannose | 647 | C_6_H_12_O_6_ |
| 20.80 | 0.19 | Methyl 5-(2-undecylcyclopropyl)pentanoate | 601 | C_20_H_38_O_2_ |
| 21.09 | 0.29 | 2,3,4,5-tetrahydroxypentanal | 677 | C_5_H_10_O_5_ |
| 22.22 | 0.22 | 3,6-Dimethyl-2-octanone | 673 | C_10_H_20_O |
| 22.85 | 0.23 | (Z,Z,Z)-9,12,15-Octadecatrienoic acid,  2-(acetyloxy)-1-[(acetyloxy)methyl]et  hyl ester | 698 | C_25_H_40_O_6_ |
| 23.12 | 0.50 | 1-Acetyl-16-methoxy-aspidospermidin-17-ol | 646 | C_22_H_30_N_2_O_3_ |
| 23.21 | 0.43 | R-Limonene | 700 | C_10_H_16_O_3_ |
| 24.51 | 0.42 | 17-Octadecynoic acid | 719 | C_18_H_32_O_2_ |
| 24.73 | 0.37 | (3á)-Cholest-5-en-3-ol | 647 | C_27_H_46_O |
| 24.79 | 0.16 | 5à-Androstan-16-one, cyclic ethylene mercaptole | 704 | C_21_H_34_S_2_ |
| 25.36 | 0.56 | 4-(2,2-Dimethyl-6-methylenecyclohe  xyl)butanal | 726 | C_13_H_22_O |
| 26.03 | 14.08 | Methyl palmitate | 919 | C_17_H_34_O_2_ |
| 27.11 | 17.09 | Oleic acid | 837 | C_18_H_34_O_2_ |
| 28.43 | 1.42 | Benzyl (6Z,9Z,12Z)-6,9,12-octadecatrienoate | 745 | C_25_H_36_O_2_ |
| 28.87 | 0.23 | 7-Oxabicyclo[4.1.0]heptane,  1-methyl-4-(2-methyloxiranyl)- | 704 | C_10_H_16_O_2_ |
| 29.06 | 8.99 | Methyl 12,13-tetradecadienoate | 824 | C_15_H_26_O_2_ |
| 29.24 | 19.39 | Methyl oleate | 863 | C_19_H_36_O_2_ |
| 29.69 | 0.54 | Icosapentaenoic acid | 623 | C_20_H_30_O_2_ |
| 29.80 | 1.86 | Methyl dihydrohydnocarpate | 766 | C_12_H_22_O_2_ |
| 30.27 | 15.65 | 9-Octadecenal | 849 | C_18_H_34_O |
| 30.71 | 2.23 | Hydnocarpic acid | 772 | C_16_H_28_O_2_ |
| 31.70 | 1.08 | N-[5-hydroxy-n-pentyl]-Arachidonic amide | 705 | C_25_H_43_NO_2_ |
| 32.05 | 0.22 | 1-Vinyl-2-(1'-ethynyl-1'-hydroxyethyl)cyclohexane | 678 | C_12_H_18_O |
| 32.11 | 0.19 | 11-Tetradecyn-1-ol | 686 | C_14_H_26_O |
| 32.26 | 0.39 | Methyl (10E)-10-heptadecen-8-ynoate | 653 | C_18_H_30_O_2_ |
| 32.46 | 1.59 | 2,3-Bis(acetyloxy)propyl (9E,12E,15E)-9,12,15-octadecatrienoate | 673 | C_25_H_40_O_6_ |
| 32.77 | 0.68 | Butyl 6,9,12-hexadecatrienoate | 651 | C_20_H_34_O_2_ |
| 33.26 | 0.80 | Tridecanedial | 656 | C_13_H_24_O_2_ |
| 33.58 | 0.13 | *cis*-*p*-Mentha-1(7),8-dien-2-ol | 715 | C_10_H_16_O |
| 34.07 | 0.19 | Undec-10-ynoic acid, butyl ester | 648 | C_15_H_26_O_2_ |
| 34.14 | 0.27 | (Z,Z)-9,12-Octadecadienoyl chloride | 656 | C_18_H_31_ClO |
| 34.18 | 0.18 | Panaxydol | 694 | C_17_H_24_O_2_ |
| 34.25 | 0.82 | 1-Methyl-4-(2-methyloxiranyl)-7-oxabicyclo[4.1.0]heptane | 677 | C_10_H_16_O_2_ |
| 34.51 | 0.17 | Caryophyllene oxide | 738 | C_15_H_24_O |
| 34.70 | 0.58 | 3-Hydroxy-, (3á,17á)-Spiro[androst-5-ene-17,1'-cyclobutan]-2'-one | 780 | C_22_H_32_O_2_ |
| 35.57 | 0.80 | 3á-Hydroxyguaia-4(15),10(14),11(13  )-trien-6,12-olide 8-(à,á-dihydroxybutyrate) | 708 | C_19_H_24_O_7_ |
| 36.31 | 0.92 | 6-Chloro-n-ethyl-1,3,5-triazine-2,4-diamine | 734 | C_5_H_8_ClN_5_ |
| 38.76 | 1.92 | 12-Methyl-E,E-2,13-octadecadien-1-  ol | 686 | C_19_H_36_O |

Table S2. The GC-MS analysis of the chemical compounds from the ethanol extract of *Nicotiana glauca* leaves

| RT | Area % | Compound | Match factor | Molecular Formula |
| --- | --- | --- | --- | --- |
| 5.25 | 0.51 | *cis*-Cyclobutane-1,2-dicarboxylic acid | 898 | C_6_H_8_O_4_ |
| 5.48 | 1.41 | 3-Methylvaleric acid | 679 | C_6_H_12_O_2_ |
| 15.03 | 1.33 | 5-Hydroxy-4-hydroxymethyl-1-(1-hydroxy-1-isopropyl)cyclohex-3-ene | 682 | C_10_H_18_O_3_ |
| 15.14 | 0.54 | 4-Cyclooctene-1-carboxaldehyde | 750 | C_9_H_14_O |
| 15.20 | 0.59 | 3-Cyclohex-3-enyl-propionic acid | 686 | C_9_H_14_O_2_ |
| 15.28 | 0.27 | D-Fructose, diethyl mercaptal, pentaacetate | 682 | C_20_H_32_O_10_S_2_ |
| 16.11 | 0.45 | Sericealactone |  |  |
| 16.29 | 0.42 | 7-Keto-8-aminopelargonic acid | 794 | C_9_H_17_NO_3_ |
| 16.76 | 0.54 | 3-Hydroxy-5-isopropyl-2-methylbenzo-1,4-quinone | 756 | C_10_H_12_O_3_ |
| 16.88 | 0.88 | 1b,5,5,6a-Tetramethyl-octahydro-1-ox  a-cyclopropa[a]inden-6-one | 724 | C_13_H_20_O_2_ |
| 18.67 | 0.54 | 2-Nitro-1-decen-4-yne | 702 | C_10_H_15_NO_2_ |
| 19.11 | 1.19 | Methyl 8,11-octadecadiynoate | 703 | C_19_H_30_O_2_ |
| 19.89 | 11.44 | Anabasine | 708 | C_10_H_14_N_2_ |
| 20.92 | 0.29 | (E)-10-Heptadecen-8-ynoic acid, methyl ester | 628 | C_18_H_30_O_2_ |
| 21.74 | 0.29 | 2-Decanynoic acid | 693 | C_10_H_16_O_2_ |
| 21.90 | 0.20 | 7-Oxabicyclo[4.1.0]heptane, 3-oxiranyl- | 738 | C_8_H_12_O_2_ |
| 21.94 | 0.23 | 2-Cyclopropyl-2-methyl-spiro[2.2]pentane-1-carboxylic acid | 698 | C_10_H_14_O_2_ |
| 22.24 | 1.36 | 1-Cyclohexene-1-methanol | 728 | C_7_H_12_O |
| 22.32 | 1.54 | 3-oxiranyl-7-Oxabicyclo[4.1.0]heptane | 784 | C_8_H_12_O_2_ |
| 23.12 | 1.56 | 5à-Androstan-16-one, cyclic ethylene mercaptole | 709 | C_21_H_34_S_2_ |
| 24.43 | 6.76 | Hexahydrofarnesyl acetone | 784 | C_18_H_36_O |
| 24.52 | 0.55 | (E)-2-Decen-1-ol | 734 | C_10_H_20_O |
| 25.35 | 0.29 | 1-Oxacyclopropyl-3,4-epoxycyclohexane | 740 | C_8_H_12_O_2_ |
| 26.02 | 1.30 | Cyclopentaneundecanoic acid methyl ester | 771 | C_17_H_32_O_2_ |
| 26.61 | 0.38 | 15(R),19(R)-Hydroxyprostaglandin E1 | 712 | C_20_H_34_O_6_ |
| 27.36 | 10.96 | Oleic acid | 774 | C_18_H_34_O_2_ |
| 27.49 | 11.29 | Palmitic acid | 772 | C_16_H_32_O_2_ |
| 28.90 | 1.05 | 1-Hexadecanol, acrylate | 810 | C_19_H_36_O_2_ |
| 29.07 | 0.80 | Methyl (4E)-4-nonenoate | 702 | C_10_H_18_O_2_ |
| 29.24 | 1.49 | 7-Nonenoic acid, methyl ester | 784 | C_10_H_18_O_2_ |
| 29.63 | 1.39 | Isophytol | 713 | C_20_H_40_O |
| 30.38 | 8.34 | Hydnocarpic acid | 830 | C_16_H_28_O_2_ |
| 30.42 | 5.63 | Phytosphingosine | 819 | C_18_H_39_NO_3_ |
| 30.90 | 1.98 | Dodecanoic acid | 705 | C_12_H_24_O_2_ |
| 31.71 | 0.47 | (Z,Z)-3,6-Nonadienal | 741 | C_9_H_14_O |
| 32.16 | 0.60 | (2-Aminoethyl)sulfanyl]sulfonic acid | 670 | C_2_H_7_NO_3_S_2_ |
| 33.16 | 0.46 | 5-Nitro-1-pentene | 765 | C_5_H_9_NO_2_ |
| 33.24 | 0.26 | 3-Ethyl-5-(2-ethylbutyl)-octadecane | 663 | C_26_H_54_ |
| 33.42 | 0.75 | Guanidineacetic acid | 773 | C_3_H_7_N_3_O_2_ |
| 34.03 | 0.36 | M-Bis(3,4-diamino-2,5-diphenyl-6-pyridino)benzene | 956 | C_40_H_32_N_6_ |
| 35.90 | 0.37 | 9,9-Dimethoxybicyclo[3.3.1]nonane-2,4-dione | 712 | C_11_H_16_O_4_ |
| 36.10 | 0.29 | Bretazenil | 766 | C_47_H_50_N_4_O_6_ |
| 36.19 | 0.24 | 2,4-Hexadien-1-ol | 664 | C_6_H_10_O |
| 36.34 | 2.03 | Tetrahydrocannabihexol | 749 | C_22_H_32_O_2_ |
| 38.74 | 0.30 | *N*-Acetylserine | 712 | C_5_H_9_NO_4_ |
| 38.85 | 0.23 | *cis*-8,11,14-Eicosatrienoic acid | 676 | C_20_H_34_O_2_ |
| 38.91 | 0.19 | 2-Aminoethanethiolsulfuric acid | 683 | C_2_H_7_NO_3_S_2_ |
| 39.01 | 0.83 | 2-Methyl-1-hexadecanol | 679 | C_17_H_36_O |
| 40.87 | 4.31 | *tert*-Hexadecanethiol | 651 | C_16_H_34_S |
| 41.30 | 0.48 | Undec-10-ynoic acid, decyl ester | 677 | C_21_H_38_O_2_ |
| 44.62 | 3.74 | Atrazine deisopropyl | 705 | C_5_H_8_ClN_5_ |
| 44.78 | 0.30 | 2-Nitrohept-2-en-1-ol | 728 | C_7_H_13_NO_3_ |
| 44.85 | 1.98 | 4-t-Butyl-2-(1-methyl-2-nitroethyl)c  yclohexanone | 709 | C_13_H_23_NO_3_ |
| 45.05 | 0.25 | 2-Hexenal | 751 | C_6_H_10_O |
